# Cardioprotective Effect of Flibanserin against Isoproterenol-Induced Myocardial Infarction in Female Rats: Role of Cardiac 5-HT2A Receptor Gene/5-HT/Ca^2+^ Pathway

**DOI:** 10.3390/ph16040502

**Published:** 2023-03-28

**Authors:** Mohamed I. Ahmed, Heba M. A. Abdelrazek, Yasser M. Moustafa, Samar Z. Alshawwa, Maysa A. Mobasher, Basel A. Abdel-Wahab, Fathy Elsayed Abdelgawad, Dina M. Khodeer

**Affiliations:** 1 Medical Administration, Ismailia 41522, Egypt; 2 Department of Physiology, Faculty of Veterinary Medicine, Suez Canal University, Ismailia 41522, Egypt; 3 Pharmacology and Toxicology Department, Faculty of Pharmacy, Badr University in Cairo, Cairo 11829, Egypt; 4 Department of Pharmacology and Toxicology, Faculty of Pharmacy, Suez Canal University, Ismailia 41522, Egypt; 5 Department of Pharmaceutical Sciences, College of Pharmacy, Princess Nourah bint Abdulrahman University, P.O. Box 84428, Riyadh 11671, Saudi Arabia; 6 Department of Pathology, Biochemistry Division, College of Medicine, Jouf University, Sakaka 72388, Saudi Arabia; 7 Department of Pharmacology, College of Pharmacy, Najran University, Najran 55461, Saudi Arabia; 8 Medical Biochemistry Department, Faculty of Medicine, Al-Azhar University, Cairo 11651, Egypt; 9 Chemistry Department, Faculty of Science, Islamic University of Madinah, Madinah 42351, Saudi Arabia

**Keywords:** flibanserin, isoproterenol, myocardial infarction, cardioprotection, 5-HT2A receptors, rats

## Abstract

Myocardial infarction (MI) is a life-threatening ischemic disease and is one of the leading causes of morbidity and mortality worldwide. Serotonin (5-HT) release during myocardial ischemia plays an important role in the progression of myocardial cellular injury. This study was conducted to investigate the possible cardioprotective effect of flibanserin (FLP) against isoproterenol (ISO)-induced MI in rats. Rats were randomly divided into five groups and were treated orally (p.o.) with FLP (15, 30, and 45 mg/kg) for 28 days. ISO was administered subcutaneously (S.C.) (85 mg/kg) on the 27th and 28th days to induce MI. ISO-induced myocardial infarcted rats exhibited a significant increase in cardiac markers, oxidative stress markers, cardiac and serum 5-HT levels, and total cardiac calcium (Ca^2+^) concentration. ISO-induced myocardial infarcted rats also revealed a remarkable alteration of electrocardiogram (ECG) pattern and significantly upregulated expression of the 5-Hydroxytryptamine 2A (5-HT2A) receptors gene. Moreover, ISO-induced myocardial infarcted rats showed significant histopathological findings of MI and hypertrophic signs. However, pretreatment with FLP significantly attenuated the ISO-induced MI in a dose-dependent manner, as the effect of FLP (45 mg/kg) was more pronounced than that of the other two doses, FLP (15 and 30 mg/kg). The present study provides evidence for the cardioprotective efficacy of FLP against ISO-induced MI in rats.

## 1. Introduction

Myocardial infarction (MI), commonly known as a heart attack, is a serious medical condition that occurs when the blood supply to the heart is blocked [1]. This blockage is usually caused by a buildup of plaque in the coronary arteries, which can lead to a complete or partial blockage of the artery [2]. When this happens, the heart muscle is deprived of oxygen and nutrients, leading to tissue death. One of the most frequent causes of morbidity and mortality worldwide, MI is primarily brought on by an imbalance between the heart’s need for oxygenated blood and its ability to deliver it as a result of coronary artery obstruction [3,4]. The heart has a limited ability for anaerobic metabolism and cannot cope with the lack of blood, nutrients, and oxygen during MI [5], leading to pathological changes and eventually resulting in cardiac dysfunction [6].

Isoproterenol (ISO) is a synthetic catecholamine and a β-adrenergic receptor agonist. Subcutaneous (S.C.) injection of ISO causes irreversible cellular damage and eventually MI in rats [7,8]. The acute electrocardiographic and hemodynamic alterations that occur in ISO-induced MI are quite similar to those that occur in patients with MI. Therefore, the rat model of ISO-induced MI offers a reliable non-invasive technique for studying the effects of various potential cardioprotective agents [9].

In the cardiac tissues, serotonin (5-HT) has been identified in platelets of the vascular beds, mast cells, and sympathetic nerve endings [10]; 5-HT is released from these compartments—mainly platelets—during myocardial ischemia, acute inflammation, and tissue damage [11,12] and plays an important role in the progression of myocardial cellular injury through various pathways utilizing 5-HT2A and 5-HT2B receptors along with uptake transporters of 5-HT [10,13,14,15]. It was previously reported the effectiveness of sarpogrelate, a selective 5-HT2A receptor antagonist, against ischemic cellular damage/ infarction size in rabbit hearts and MI in rats [10,16,17] and blockade of 5-HT2 receptors by ketanserin and cinanserin resulted in the protection of the isolated rat heart against ischemia in terms of cardiac function [18].

Flibanserin (FLP) is a drug that has been approved by the FDA for the treatment of premenopausal women with hypoactive sexual desire disorder (HSDD) [19]. HSDD is a condition characterized by a lack of interest in sexual activity that causes distress or interpersonal difficulty. FLP is the first drug approved for the treatment of HSDD in women [20]. FLP works by targeting the brain’s 5-HT, norepinephrine (NE), and dopamine (DA) systems, which are involved in sexual desire. It is thought to alter the level of these neurotransmitters, which can lead to an increase in sexual desire. FLP is taken once daily, and it is recommended that women take it at bedtime [21]. FLP is a non-hormonal drug chemically described as N-alkylpiperazine derivative used to treat acquired generalized HSDD in premenopausal women [22]. FLP has a high affinity for 5-HT post-synaptic 5-HT1A and 5-HT2A receptors, displaying agonist activity on 5-HT1A receptors and antagonist activity on 5-HT2A receptors, after the launching of FLP in the market worldwide. Premenopausal women who predominantly suffer from cardiovascular disease started medication with it. The effect of FLP in developing or treating cardiac symptoms is still unknown. So, the present study was designed to investigate the potential cardioprotective effect of FLP against ISO-induced MI in female Wistar rats for the first time.

## 2. Results

### 2.1. Effect of FLP Pretreatment on Heart Rate (HR), R Wave Amplitude, ST Segment Amplitude, and Qt Interval

**Figure 1:**
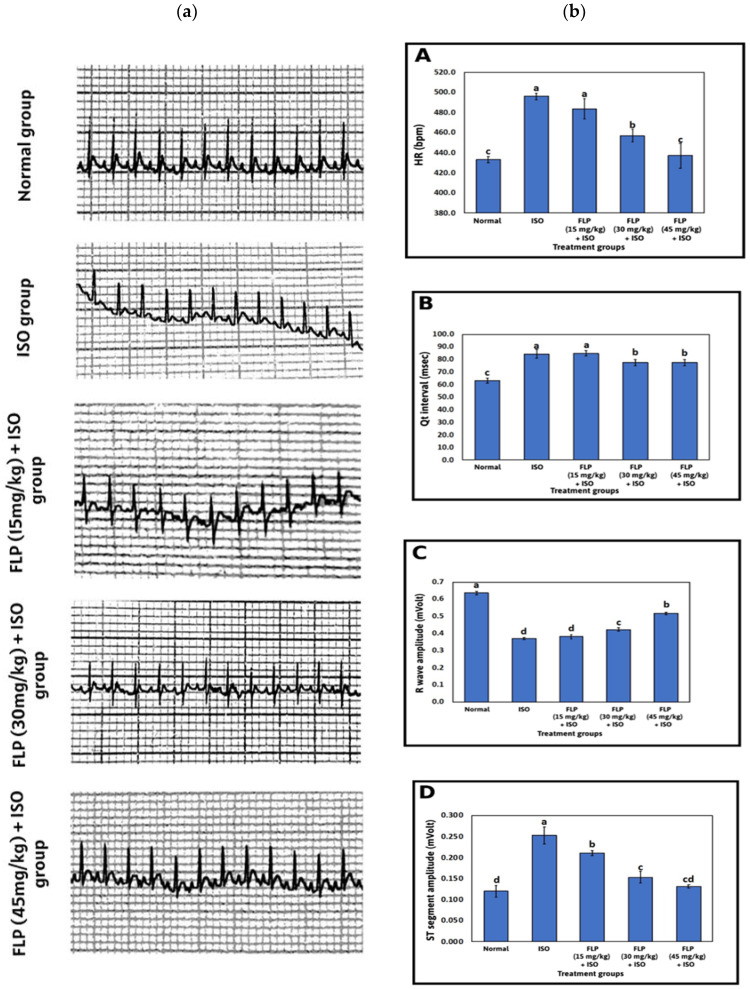
The ECG examination in different treated groups. (**a**): The electrocardiographic effect of FLP in different doses on ISO-induced MI in rats. (**b**): ECG in experimental rats pretreated with FLP (15, 30, and 45 mg/kg) in an ISO-induced MI model on HR, QT interval, R amplitude, and ST segment amplitude. (**A**) Normal group, (**B**) Iso group, (**C**) FLB 15 mg + Iso group, (**D**) FLB 30 mg + Iso group and FLB 45 mg + Iso group. Bars with different letters are significantly different according to DMRT at *p* < 0.001 level (*n* = 5). Data are expressed as Mean ± SD. ECG = electrocardiogram, FLP = flibanserin, ISO = isoproterenol, MI = myocardial infarction, HR = heart rate, DMRT = Duncan’s multiple range test.

As shown in Figure 1a, both the ISO group and FLP (15 mg/kg) group showed a significant increase in HR when compared to the normal group (*p* < 0.001). In contrast, rats pretreated with FLP (30 and 45 mg/kg) showed a significant decrease in HR when compared to the ISO group and FLP (15 mg/kg) group (*p* < 0.001). A higher dose of FLP (45 mg/kg) decreased HR significantly to a much greater extent when compared to a lower dose (FLP 30 mg/kg) (*p* < 0.001) (Figure 1a).

Both the ISO group and FLP (15 mg/kg) group showed a significant increase in Qt interval when compared to the normal group (*p* < 0.001). In contrast, rats pretreated with FLP (30 and 45 mg/kg) showed a significant decrease in Qt interval when compared to the ISO group and FLP (15 mg/kg) group (*p* < 0.001) (Figure 1a,b).

Both the ISO group and FLP (15 mg/kg) group showed a significant decrease in R wave amplitude when compared to the normal group (*p* < 0.001). In contrast, rats pretreated with FLP (30 and 45 mg/kg) showed a significant increase in R wave amplitude when compared to the ISO group and FLP (15 mg/kg) group (*p* < 0.001). A higher dose of FLP (45 mg/kg) increased R wave amplitude significantly to a much greater extent when compared to a lower dose (FLP 30 mg/kg) (*p* < 0.001) (Figure 1a,b).

The ISO group showed a significant elevation in ST segment amplitude when compared to the normal group (*p* < 0.001). In contrast, rats pretreated with FLP (15, 30, and 45 mg/kg) showed a significant decrease in ST segment amplitude when compared to the ISO group (*p* < 0.001). Higher doses of FLP (30 and 45 mg/kg) decreased ST segment amplitude significantly to a much greater extent when compared to a lower dose (FLP 15 mg/kg) (*p* < 0.001) (Figure 1a,b).

Effect of FLP pretreatment on myocardial final body weight, heart weight, left ventricular weight, myocardial weight index, and left ventricle weight index.

Both the ISO group and FLP (15 mg/kg) group showed a significant increase in myocardial weight index when compared to the normal group (*p* = 0.001). In contrast, rats pretreated with FLP (30 and 45 mg/kg) showed a significant decrease in myocardial weight index when compared to the ISO group (*p* = 0.001) (Table 1).

Both the ISO group and FLP (15 mg/kg) group showed a significant increase in the left ventricle weight index when compared to the normal group (*p* < 0.001). In contrast, rats pretreated with FLP (30 and 45 mg/kg) showed a significant decrease in left ventricle weight index when compared to the ISO group and FLP (15 mg/kg) group (*p* < 0.001) (Table 1).

### 2.2. Effect of FLP Pretreatment on Histopathology

**Figure 2:**
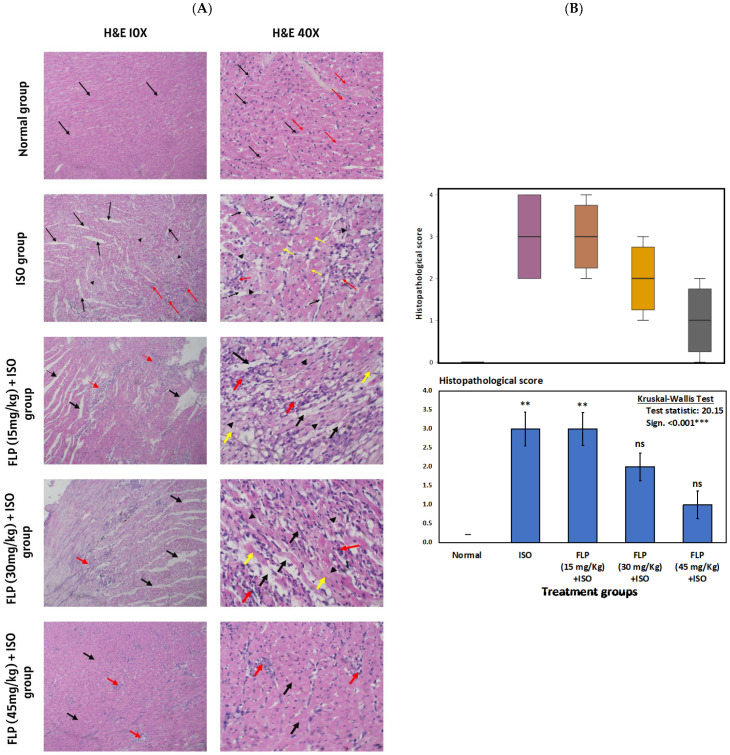
Histopathological examination in the different treated groups. (**A**): Photomicrograph for specimens from the heart stained with H&E 10× & 40× in different treated groups. In the normal group: [H&E 10×] shows Compact, uniformly arranged myocardial fibers (Black arrows), [H&E 40×] myocytes show preserved uniform nuclei (Black arrows) and cytoplasmic cross striation (Red arrows). In ISO group: [H&E 10×] shows a wide separation of myocardial fibers due to interstitial edema (Black arrows), areas of myocardial fibers loss of eosinophilic staining and becoming pallor in appearance (Arrowheads), the influx of inflammatory cells into myocardial fibers (Red arrows), [H&E 40×] shows pathological changes of MI, interstitial edema between myocardial fibers (Black arrows), loss of cytoplasmic striation of myocytes with shrinkage of nuclei and prominence of the cell membrane (Arrowheads), some myocytes show an absence of nuclei (Yellow arrows), the influx of macrophages, indicating an inflammatory response to remove dead fibers (Red arrows). In FLP (15 mg/kg) + ISO group: [H&E 10×] shows no significant difference when compared to the ISO group as there is still a wide separation of myocardial fibers due to interstitial edema (Black arrows), the influx of inflammatory cells into myocardial fibers (Red arrows), [H&E 40×] shows persistent pathological changes of MI, interstitial edema between myocardial fibers (Black arrows), loss of cytoplasmic striation of myocytes with shrinkage of nuclei and prominence of the cell membrane (Arrowheads), some myocytes show an absence of nuclei (Yellow arrows), the influx of macrophages indicating an inflammatory response to remove dead fibers (Red arrows). In FLP (30 mg/kg) + ISO group: [H&E 10×] still shows interstitial edema (Black arrows); however, a slight reduction in inflammatory cell influx is noticed (Red arrow), [H&E 40×] shows persistent pathological changes of MI, yet to a milder degree: interstitial edema between myocardial fibers (Black arrows), loss of cytoplasmic striation of few myocytes with shrinkage of nuclei and prominence of the cell membrane (Arrowheads), absence of nuclei in few myocytes (Yellow arrows), reduction in macrophages influx (Red arrows). In FLP (45 mg/kg) + ISO group: [H&E 10×] shows compact uniformly arranged myocardial fibers and absence of interstitial edema (Black arrows); few areas of chronic inflammatory cells are seen (Red arrow), [H&E 40×] myocytes show uniform nuclei and eosinophilic cytoplasm (Black arrows), few macrophages are seen (Red arrows). (**B**): histopathologic score in experimental rats pretreated with FLP (15, 30, and 45 mg/kg) in an ISO-induced MI model. Data were analyzed using Kruskal-Wallis followed by Dunn’s test for multiple comparisons against the normal group at *p* < 0.05., ** *p* < 0.01, *** *p* < 0.001, *n* = 6. H&E = hematoxylin and eosin, FLP = flibanserin, ISO = isoproterenol, MI = myocardial infarction.

Histopathological examination of the normal group showed compact, uniformly arranged myocardial fibers with preserved uniform nuclei and cytoplasmic cross striation, while the ISO group and FLP (15 mg/kg) group revealed wide separation of myocardial fibers due to interstitial edema, the influx of inflammatory cells into myocardial fibers, loss of cytoplasmic striation of myocytes with shrinkage of nuclei, and prominence of the cell membrane. FLP (30 mg/kg) group revealed persistent pathological changes of MI yet to a milder degree, while FLP (45 mg/kg) group revealed the nearly normal architecture of myocardial fibers with a mild degree of inflammatory infiltrate (Figure 2A,B).

### 2.3. Effect of FLP Pretreatment on Serum Troponin I (TnI), Lactate Dehydrogenase (LDH), Creatinine Kinase-MB (CK-MB), and Creatine Kinase (CK)

**Figure 3:**
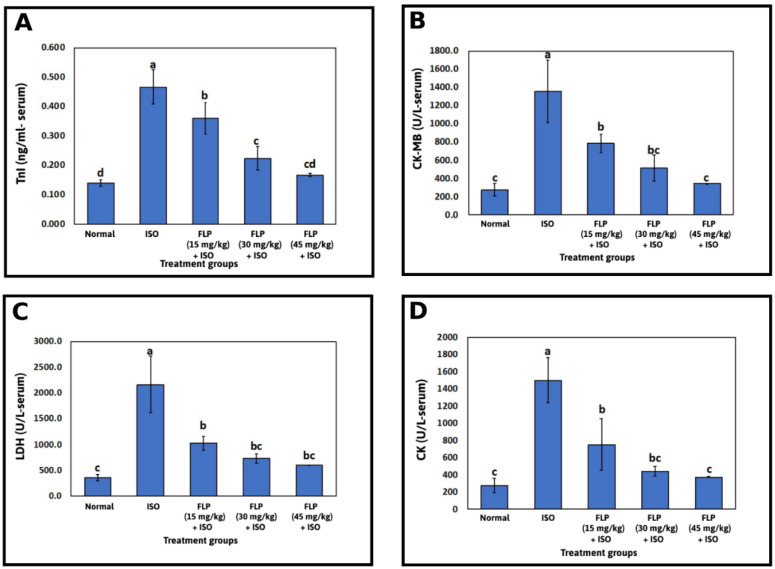
Serum cardiac enzymes levels in experimental rats pretreated with FLP (15, 30, and 45 mg/kg) in an ISO-induced MI model (**A**): The cardiac serum level of Tnl (**B**): The cardiac serum level of CK-MB, (**C**): The cardiac serum level of LDH. (**D**): The cardiac serum level of CK. Bars with different letters are significantly different according to DMRT at *p* < 0.001 level (*n* = 5). Data are expressed as Mean ± SD. FLP = flibanserin, ISO = isoproterenol, MI = myocardial infarction, Tnl = troponin I, CK-MB = creatinine kinase-MB, LDH = lactate dehydrogenase, CK = creatine kinase, DMRT = Duncan’s multiple range test.

The ISO group showed a significant elevation in serum TnI concentration when compared to the normal group (*p* < 0.001). In contrast, rats pretreated with FLP (15, 30, and 45 mg/kg) showed a significant decline in serum TnI concentration when compared to the ISO group (*p* < 0.001). Higher doses of FLP (30 and 45 mg/kg) decreased serum TnI concentration significantly to a much greater extent when compared to a lower dose (FLP 15 mg/kg) (*p* < 0.001) (Figure 3A).

The ISO group a showed significant elevation in Serum CK-MB level when compared to the normal group (*p* < 0.001). In contrast, rats pretreated with FLP (15, 30, and 45 mg/kg) showed a significant decline in Serum CK-MB level when compared to the ISO group (*p* < 0.001). The higher dose of FLP (45 mg/kg) decreased serum CK-MB level significantly to a much greater extent when compared to the lower dose (FLP 15 mg/kg) (*p* < 0.001) (Figure 3B).

The ISO group showed a significant elevation in the Serum LDH level when compared to the normal group (*p* < 0.001). In contrast, compared to the ISO group, rats pretreated with FLP (15, 30, and 45 mg/kg) demonstrated a significant decrease in serum LDH levels. (*p* < 0.001) (Figure 3C). The ISO group showed a significant elevation in Serum CK level when compared to the normal group (*p* < 0.001). In contrast, rats pretreated with FLP (15, 30, and 45 mg/kg) showed a significant decline in Serum CK level when compared to the ISO group (*p* < 0.001). The higher dose of FLP (45 mg/kg) decreased serum CK level significantly to a much greater extent when compared to the lower dose (FLP 15 mg/kg) (*p* < 0.001) (Figure 3D).

### 2.4. Effect of FLP Pretreatment on Cardiac Oxidative Stress Markers

**Figure 4:**
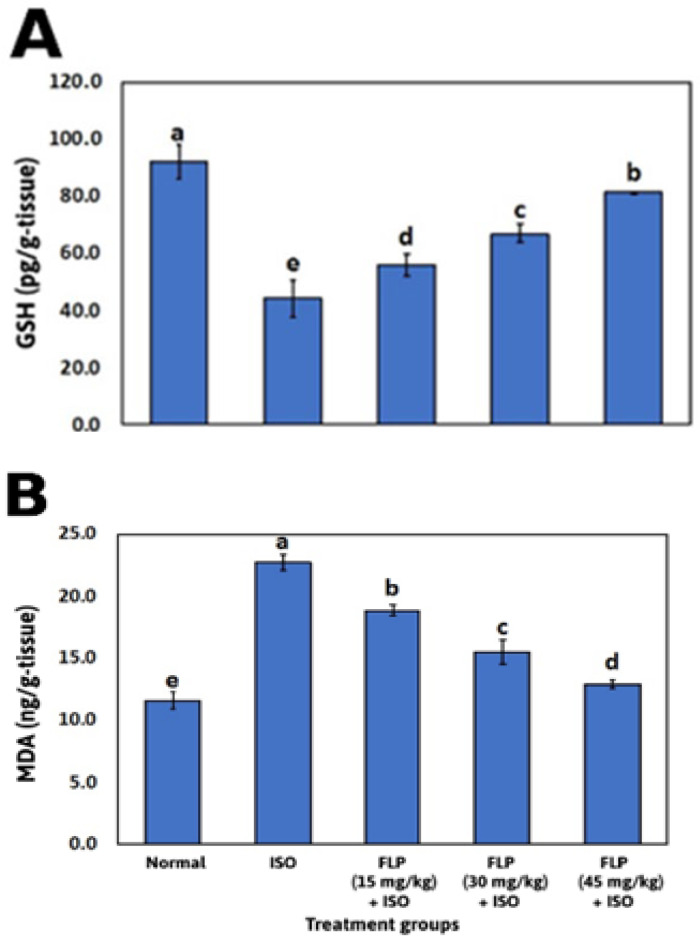
Effect of FLP in different doses on heart tissue level of GSH and MDA. (**A**): Heart level of GSH in experimental rats pretreated with FLP (15, 30, and 45 mg/kg) in an ISO-induced MI model. (**B**) Heart level of MDA in experimental rats pretreated with FLP (15, 30, and 45 mg/kg) in an ISO-induced MI model. Bars with different letters are significantly different according to DMRT at *p* < 0.001 level (*n* = 5). Data are expressed as Mean ± SD. FLP = flibanserin, GSH = reduced glutathione, MDA = malondialdehyde, ISO = isoproterenol, DMRT = Duncan’s multiple range test.

The ISO group showed a significant decline in cardiac reduced glutathione (GSH) concentration when compared to the normal group (*p* < 0.001). In contrast, rats pretreated with FLP (15, 30, and 45 mg/kg) showed a significant elevation in cardiac GSH concentration when compared to the ISO group (*p* < 0.001). Increasing the dose of FLP resulted in an increase in cardiac GSH concentration significantly to a much greater extent (*p* < 0.001) (Figure 4A).

The ISO group showed a significant elevation in cardiac malondialdehyde (MDA) concentration when compared to the normal group (*p* < 0.001). In contrast, rats pretreated with FLP (15, 30, and 45 mg/kg) showed a significant decline in cardiac MDA concentration when compared to the ISO group (*p* < 0.001). Increasing the dose of FLP resulted in a decrease in cardiac MDA concentration significantly to a much greater extent (*p* < 0.001) (Figure 4B).

### 2.5. Effect of FLP Pretreatment on Serum 5-HT, Cardiac 5-HT, and Cardiac Total Calcium (Ca^2+^)

The level of serum 5-HT significantly dropped in the ISO group. When compared to the normal group (*p* < 0.001). In contrast, rats pretreated with FLP (15, 30, and 45 mg/kg) showed a significant elevation in serum 5-HT level when compared to the ISO group (*p* < 0.001). Increasing the dose of FLP resulted in an increase in serum 5-HT level significantly to a much greater extent (*p* < 0.001) (Table 2).

The ISO group showed a significant decline in cardiac 5-HT levels when compared to the normal group (*p* < 0.001). In contrast, rats pretreated with FLP (15, 30, and 45 mg/kg) showed a significant elevation in cardiac 5-HT level when compared to the ISO group (*p* < 0.001). Increasing the dose of FLP resulted in an increase in cardiac 5-HT level significantly to a much greater extent (*p* < 0.001) (Table 2).

The ISO group showed a significant elevation in cardiac total Ca^2+^ concentration when compared to the normal group (*p* < 0.001). In contrast, rats pretreated with FLP (15, 30, and 45 mg/kg) showed a significant decline in cardiac total Ca^2+^ concentration when compared to the ISO group (*p* < 0.001). A higher dose of FLP (45 mg/kg) decreased cardiac total Ca^2+^ concentration significantly to a much greater extent when compared to lower doses (FLP 15 and 30 mg/kg) (*p* < 0.001) (Table 2).

### 2.6. Effect of FLP Pretreatment on Cardiac 5-HT2A Gene Expression

The ISO group showed a significant elevation in cardiac 5-HT2A receptors normalized fold change when compared to the normal group (*p* < 0.001). In contrast, rats pretreated with FLP (15, 30, and 45 mg/kg) showed a significant decline in cardiac 5-HT2A receptors normalized fold change when compared to the ISO group (*p* < 0.001). A higher dose of FLP (45 mg/kg) decreased cardiac 5-HT2A receptors normalized fold change significantly to a much greater extent when compared to a lower dose (FLP 15 mg/kg) (*p* < 0.001) (Table 3).

## 3. Discussion

Cardiovascular illnesses are the main cause of death [23]. The most notable of these illnesses is MI, which permanently damages heart tissue [24]. It increases the death rate among those who are impacted by it [25]. Numerous processes, including oxidative stress, Ca^2+^ overload, endothelial and cardiac damage, contractile dysfunction, and cell death by necrosis or apoptosis, or both, are combined in the pathogenesis of this disease [26]. ISO is a synthetic catecholamine and a β-adrenergic receptor agonist. S.C. injection of ISO causes irreversible cellular damage and eventually MI in rats [7,8]. A reliable non-invasive method for examining the effects of numerous potentially cardioprotective drugs is available in the rat model of ISO-induced MI [9].

Mast cells, sympathetic nerve terminals, and platelets from vascular beds have all been found to contain 5-HT in cardiac tissues [10]. Further, 5-HT is released from these compartments during cardiac ischemia, acute inflammation, and tissue injury, primarily from platelets-HT is released from these compartments during cardiac ischemia, acute inflammation, and tissue injury, primarily from platelets [11,12]. Platelet aggregation, thrombus formation, smooth muscle cell contraction, and coronary artery spasms are all caused by the release of additional 5-HT from the dense granules of platelets after binding of 5-HT to their 5-HT2A receptors [10,27,28]. The myocardial interstitium accumulates 5-HT, which plays a significant role in the development of cardiac cellular damage through a variety of pathways, including 5-HT2A and 5-HT2B receptors as well as uptake transporters of 5-HT [10,13,14,15].

**Figure 5:**
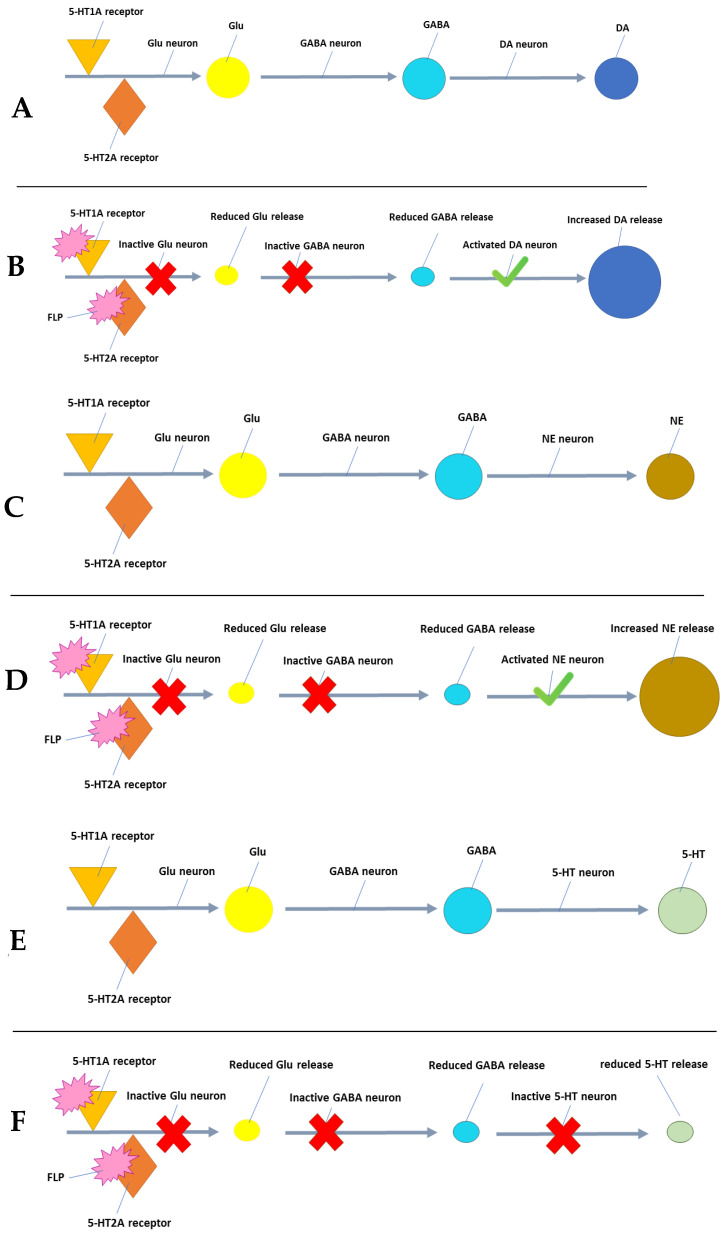
Proposed FLP action on DA, NE, and 5-HT centers of the brain. The figure represents the dopaminergic system (**A**) before and (**B**) after the administration of flibanserin. While noradrenergic system (**C**) before and (**D**) after administration of flibanserin. Finally, serotonergic system (**E**) before and (**F**) after administration of flibanserin. Glu = Glutamate, GABA = Gamma-aminobutyric acid, NE = Norepinephrine, DA = Dopamine, 5-HT = Serotonin, FLP = Flibanserin.

As an N-alkylpiperazine derivative, FLP is a non-hormonal medication used to treat acquired global HSDD in premenopausal women [29]. Furthermore, FLP exhibits agonist activity on 5-HT1A receptors and antagonist activity on 5-HT2A receptors, demonstrating a high affinity for 5-HT post-synaptic 5-HT1A and 5-HT2A receptors. Reduced glutamate (Glu) transmission to the brainstem, which in turn causes disinhibition of the ascending adrenergic and dopaminergic neurons and inhibition of the ascending serotoninergic neurons, is the result of preferential FLP activation of 5-HT1A receptors and blockade of 5-HT2A receptors on the cortical pyramidal neurons. Together, these actions increase NE and DA and transiently decrease 5-HT, restoring an appropriate balance of excitatory and inhibitory activity of the brain reward centers to the prefrontal cortex (Figure 5) [30]. FLP may result in central nervous system (CNS) depression with drowsiness and sedation and can even cause hypotension and syncope by itself. Dry mouth, fatigue, and insomnia can also occur with FLP use [19].

The objective of the current investigation was to examine for the first time any potential cardioprotective effects of various FLP dosages (15, 30, 45 mg/kg) against ISO-induced MI in female rats. This aim was based on previous studies which demonstrated the effectiveness of sarpogrelate, a selective 5-HT2A receptor antagonist, against ischemic cellular damage/ infarction size in rabbit hearts and MI in rats [10,16,17]; the isolated rat heart was protected against ischemia in terms of cardiac function by ketanserin and cinanserin’s inhibition of 5-HT2 receptors [18], concluding that inhibition of the 5-HT system by FLP may have cardioprotective effect in ISO-induced MI.

Reactive oxygen species (ROS) are produced in excess, antioxidant defenses are depleted, and the development of an oxidative stress state is all associated with MI, which triggers a chain of pathological events causing functional and structural damage to cardiomyocytes [31]. Massive amounts of ROS are produced by ISO auto-oxidation, which can attack any kind of molecule, but their main target seems to be polyunsaturated fatty acids in membranes. This causes the formation of peroxyl radicals, which then attack nearby fatty acids in membranes to start a chain reaction that results in lipid peroxidation [32,33]. Lipid peroxidation is a major pathogenic event in myocardial necrosis, and the accumulation of lipid hydroperoxides reflects cardiac constituents’ damage [34]. MDA is considered a major lipid peroxidation end product; increased MDA content can contribute to the increased generation of free radicals and/or decreased activities of the antioxidant defense system [35].

By observable changes in oxidative stress markers, such as a considerable rise in MDA concentration, the incidence of oxidative stress in the ISO group was validated in the current investigation. MDA is a critical indicator of MI pathogenesis [36]. This could be a result of high ROS generation by ISO autooxidation [37]. Additionally, there was a considerable drop in the non-enzymatic antioxidant biomarker GSH, which is consistent with earlier research [31]. However, FLP pretreated groups were found to significantly attenuate the oxidative stress, demonstrated by the significant decrease in MDA concentration and the increase in GSH concentration in those groups. Lipid peroxidation inhibition with antioxidant activation is considered protection from MI [38]. These findings point to FLP’s cardioprotective ability to counteract the oxidative stress state involved in ISO-induced MI. Numerous MI diagnostic markers are present in the myocardial, and when it is injured, cytosolic enzymes leak into the bloodstream, increasing their serum levels [39]. The release of these enzymes reflects an alteration in the plasma membrane integrity and permeability [40] and indicates cell necrosis severity and ISO-mediated myocyte injury [41].

A component of the myofibril contractile apparatus known as cardiac TnI regulates the Ca^2+^-mediated interaction between actin and myosin, which results in muscle contraction. [42]. TnI is a highly specific and extremely sensitive diagnostic marker of MI and has not been isolated from skeletal muscles [43,44] and is thought to be the gold standard biomarker for diagnosing MI [45]. LDH is an enzyme responsible for anaerobic metabolism in many tissues, such as the heart and the skeletal muscles. The isoenzyme LDH1 is predominantly present in damaged and necrotic myocardium and serves as a significant biomarker of MI [46,47,48]. The enzyme CK catalyzes the reversible conversion of creatine and adenosine triphosphate (ATP) to creatine phosphate and adenosine diphosphate. The myocardial contains the dimeric enzyme, which has two subunits, M and B. About 20% of the total cardiac CK is in the MB form, providing specificity and sensitivity in the diagnosis of MI [44,46,49].

These results were consistent with earlier studies and indicated ISO-induced necrotic damage to the myocardium. In the current study, MI induced by ISO was confirmed by a significant increase in serum TnI and LDH, in addition to CK and CK-MB in the ISO group, as compared to the normal group [50,51]. These cardiac blood marker levels did, however, significantly decline in FLP treatment groups. These results imply that FLP prevents cardiac damage by maintaining the structural and functional integrity of the plasma membrane and contractile system of the myocyte and limiting the leakage of these cardiac enzymes into the circulation.

The primary criteria often employed for making a conclusive diagnosis of MI are electrocardiogram (ECG) abnormalities. One of the key indicators of cardiomyocyte membrane damage is ST segment elevation, which may indicate a difference between the ischemia and non-ischemic zones and subsequently disrupt cell membrane function [52,53]. The QT interval reflects the functional integrity of the myocardium, which is assessed by inward sodium and calcium currents and outward potassium and chloride currents during the electrical systole of the heart [54,55]. The aberrant potential of the heart, such as arrhythmias, cardiac dysfunction, and rapid cardiac collapse, is indicated by QT interval lengthening, which is linked to cardiac vagal dysfunction [53]. ECG monitoring of the ISO group showed significant ST-segment elevation, a marked decrease in R wave amplitude, along with QT interval prolongation and a significant increase in HR, confirming ISO-induced MI. ISO-induced ECG pattern alterations are in agreement with previous studies [56,57,58]. The significant increase in HR observed in the ISO group could be due to the activation of β1 adrenoceptors by ISO [56]. It has been demonstrated that the increase in HR is responsible for increasing cardiac oxygen consumption leading to accelerated myocardial necrosis [59]. The onset of myocardial edema might cause a decrease in R wave amplitude after ISO administration [59]. The current study showed that FLP-pretreated groups significantly attenuated ECG abnormalities induced by ISO.

Histopathological examination of the ISO group revealed an influx of inflammatory cells into myocardial fibers along with interstitial edema, consistent with earlier research [60]. These findings and the biochemical abnormalities confirmed the severity of the myocardial damage. A significant improvement was noticed in the FLP (45 mg/kg) group. The nearly normal architecture of myocardial fibers with a mild degree of inflammatory infiltrate in the FLP (45 mg/kg) group furtherly confirmed the cardioprotective effect of FLP.

Following ISO administration in the ISO group, the heart weight index and left ventricle weight index increased significantly as an indication of cardiac hypertrophy, which might be caused by increased water and protein content, edematous intramuscular space, necrosis of cardiac muscle fibers, and activation of Calcium/calmodulin-dependent protein kinase II/ histone deacetylase 4 (CamKII/HDAC4) pathway [56,61,62]. These results are in agreement with previous studies [56,59]. Pretreatment with FLP significantly reduced the heart weight index and left ventricle weight index, indicative of its cardioprotective ability against infiltration and cardiac damage, in addition to blockade of cardiac 5-HT2A receptors, which was shown to play a major role in cardiac hypertrophy through inhibition of the CamKII/HDAC4 pathway [62].

Calcium is a critical cytoplasmic signaling molecule in most cellular reactions. The primary organelles that store Ca^2+^ include the mitochondria and the sarcoplasmic reticulum [63]. The participation of Ca^2+^ signaling in oxidative stress and necrosis present in acute MI is well described in the literature [64]. One of the most significant fundamental causes of many types of cardiac dysfunction is thought to be the induction of Ca^2+^ influx caused by ISO, which harms the myocardium. Additionally, it has been observed that L-type Ca^2+^+ channels had a role in mediating ISO-induced cardiomyopathy by increasing intracellular Ca^2+^ concentration [65,66]. MI provides a platelet-stimulating micro-environment that leads to the release of 5-HT from platelets. 5-HT has been shown to stimulate intracellular Ca^2+^ mobilization and Ca^2+^ influx via 5-HT2A G protein-coupled receptors/IP3-dependent signaling pathway and activation of L-type Ca^2+^ channels [12,27]. Following ischemia, myocardial cells’ intracellular Ca^2+^ concentration rises, resulting in Ca^2+^ overload. Ca^2+^ overload can trigger a number of irreversible cellular damage responses, including cardiac contractile failure and apoptosis. Extracellular Ca^2+^ can enter the mitochondria in excess, causing mitochondrial Ca^2+^ overload, which decreases ATP synthesis, aggravates abnormalities of energy metabolism, and produces ROS, eventually inducing cardiac apoptosis [67,68]. Phospholipases, primarily phospholipase A and protein kinase C (PKC), can be activated by intracellular Ca^2+^ and damage the structure of the cell membrane. Additionally, the reaction generates a number of harmful chemicals, including oxygen free radicals, prostaglandins, leukotrienes, and free fatty acids [69], increasing the permeability of cell membranes, resulting in mitochondrial malfunction, and encouraging severe cardiac apoptosis. Additionally, too much Ca^2+^ can activate endonuclease, caspase, and calpain, which triggers the breakdown of intracellular proteins and lipids [70,71].

In the present study, the incidence of Ca^2+^ overload was demonstrated by a significant increase in cardiac total Ca^2+^ concentration in the ISO group. However, FLP groups were found to significantly attenuate the increase in cardiac total Ca^2+^ concentration offering cardioprotection from Ca^2+^ overload-induced injury.

Serotonin receptors are divided into seven classes based on their structural and functional characteristics (5-HT1 to 5-HT7) [72]. Due to their participation in the physiological and pathological processes in the cardiovascular system, 5-HT2 receptors are of great therapeutic relevance. In addition to being present in various areas of the CNS, 5-HT2A receptors [73] are observed on the cell membrane of platelets [74], smooth muscle cells [75], and cardiomyocytes along with 5-HT2B receptors [15,76]. 5-HT accumulates in the myocardial interstitium and plays an important role in the progression of myocardial cellular injury through various pathways utilizing 5-HT2A and 5-HT2B receptors along with uptake transporters of 5-HT [10,13,14,15]. Binding of 5-HT to platelets 5-HT2A receptors induce more 5-HT to be released from the platelet’s dense granules through a vicious cycle leading to platelets aggregation, thrombus formation, contraction of smooth muscle cells, and coronary artery spasms [10,27,28]. The activation of phospholipase C (PLC) by 5-HT2A receptors, which are connected to G q/11 proteins, results in the buildup of inositol triphosphate (IP3), diacylglycerol, and PKC [77]. Accumulation of IP3 through PLC caused hydrolysis of phosphoinositides, causing Ca^2+^ release from intracellular pools. In addition, 5-HT2A can also activate L-type Ca^2+^ channels allowing for Ca^2+^ influx, which can lead to Ca^2+^ overload and cellular damage [12,27]. In heart cells, 5-HT induces a hypertrophic response through 5-HT2A receptors. Cardiomyopathy is mostly caused by the activation of Calcium/calmodulin-dependent protein kinase II (CamKII), which is activated by Gq-coupled receptors in response to increased Ca^2+^ concentration. In addition, CamKII can phosphorylate histone deacetylase 4 (HDAC4), a downstream partner of CamKII, to promote hypertrophic gene expression and pathological cardiac remodeling [62].

A cascade of protein interactions known as the Janus kinase-signal transducers and activators of transcription (JAK-STAT) pathway is linked to immunity, cell death, cell division, and tumor development. The JAK/STAT pathway is activated by 5-HT2A receptors in vascular smooth muscle cells, which then increases the production of the Transforming growth factor (TGF). Additionally, 5-HT-mediated activation of the JAK/STAT pathway through 5-HT2A receptors is followed by the activation of monocyte chemoattractant protein-1, which in turn stimulates the production of TGF- through the activation of macrophages. Moreover, extracellular signal-regulated kinase (ERK) activation by 5-HT2A receptors, along with elevated ROS, induces the expression of TGF-β. TGF-β appears to be responsible for cardiomyocyte apoptosis, cardiac fibrosis, and cardiac hypertrophy. TGF-β levels are known to be elevated in MI, thereby aggravating the myocardial injury further [77,78]. Cells absorb the elevated myocardial interstitial 5-HT via 5-HT uptake transporters and then metabolized by monoamine oxidase (MAO) to yield 5-hydroxyindole acetic acid and hydrogen peroxide, which causes myocardial cell injury [14,79,80]. Additionally, 5-HT2A receptors are implicated in the activation and phosphorylation of ERK; during this route, decreased Nicotinamide adenine dinucleotide phosphate produces ROS as an intermediate [77]. Cardiomyocytes are protected against ischemic cellular damage via translocation of Protein kinase C-epsilon (PKC-ɛ), followed by the opening of mitochondrial ATP-sensitive potassium channel (mitoKATP) leading to a decrease in mitochondrial Ca^2+^ uptake, which prevent Ca^2+^ induced opening of mitochondrial permeability transition pore, decrease in ROS generation and swelling of the mitochondria which preserve the architecture of the intermembrane space, and improves fatty acid oxidation and ATP production [81,82,83]. It was suggested that Increased myocardial interstitial 5-HT could prevent PKC- from translocating, which would otherwise cause the mitoKATP channel to activate [10].

In the current investigation, the ISO group demonstrated a notable decline in both serum and cardiac 5-HT levels below the control level after 24 h of the second ISO dose when compared to the normal group. In FLP groups, serum and cardiac 5-HT levels increased gradually with increasing FLP dose when compared to the ISO group after 24 h of the second ISO dose. However, 5-HT levels in any of the FLP groups were still significantly lower than that of the normal group. According to previous studies [10,14], in ischemic rat and rabbit models, the myocardial interstitial 5-HT level increased during ischemia but decreased shortly after a period of reperfusion and even below the control level in the ischemic rabbit model, as increased MAO-dependent breakdown of 5-HT absorbed into cells aids in the removal of stored 5-HT.

Our hypothesis to explain our current results is that during ISO-induced MI, the 5-HT level increased in the ISO group and in the FLP groups. However, the degree of 5-HT level elevation in the FLP groups is thought to be inversely proportional to the FLP dose as a result of blocking the platelet’s 5-HT2A receptors inhibiting the release of 5-HT from it. FLP (45 mg/kg) was shown to offer the most cardioprotective effect and showed 5-HT levels closest to that of the normal group.

The current study may conclude that increasing FLP dose resulted in decreasing the extent of 5-HT release during MI induced by ISO in a dose-dependent manner and that degradation of 5-HT by MAO and subsequent clearance is furtherly augmented according to the increased 5-HT levels, which led to the decreased level of 5-HT below normal level in the ISO group and in the FLP groups after 24 h of the second ISO dose. Significantly upregulated expression of cardiac 5-HT2A gene was noticed in the ISO group when compared to the normal group; this elevated expression was markedly downregulated in the FLP groups indicating that FLP amelioration of ISO-induced MI may be, at least in part, through downregulation of 5-HT2A gene in the heart. This study demonstrated a novel cardioprotective effect of FLP against ISO-induced MI in rats in a dose-dependent manner as the increase in FLP dose (15, 30, and 45 mg/kg) showed a corresponding improvement of myocardial damage induced by ISO. FLP (45 mg/kg) was shown to offer the most cardioprotective effect.

## 4. Materials and Methods

### 4.1. Experimental Animals

Female adult Wistar rats weighing 121–185 g were used in the present study. Rats were housed in plastic cages under environmentally controlled conditions (temperature 25 ± 1 °C and normal light/dark cycle) with free access to standard food and water ad libitum. Rats were left to acclimatize for one week before starting the experiment. Animal care and experimental procedures were approved by the Institutional Research Ethics Committee at the Faculty of Pharmacy Suez Canal University, ethical approval number 201903MA1. The median lethal dose (LD50) of ISO in rats was found to be 600 mg/kg S.C. [84], and the LD50 of FLP in rats is 3.844 g/kg orally, while and maximum tolerated dose of FLP in rats is 0.058 g/kg [85], FLP dose selection was based on previous experiments [86,87,88,89].

### 4.2. Drugs and Chemicals

Flibanserin was purchased from Rameda (Giza, Egypt) and was suspended in water. ISO hydrochloride was purchased from Sigma-Aldrich (St. Louis, MO, USA) and was dissolved in sterile normal saline. All of the other chemicals utilized were of analytical grade and were obtained from El-Gomhouria Co. (Cairo, Egypt).

### 4.3. Experimental Design

#### 4.3.1. Sample Size Calculations

To evaluate the difference between the five treatment regimens, one-way ANOVA is proposed. A minimum total sample size of 30 samples will be sufficient to detect the effect size of 0.70 according to a power (1-β = 0.80) of 80% at a significance probability level of 0.05 and a partial eta squared of 0.33. According to sample size calculations, each treatment group would be represented by 6 samples. The sample size was calculated according to G*Power software version 3.1.9.6.

#### 4.3.2. Experiment

Thirty rats were randomly assigned into five groups, six rats each. The treatment regimen was as follows:

1. Normal group: Rats received two s.c. Injections of normal saline (vehicle of ISO) in a volume of 1.5 mL/kg, one injection per day on days 27 and 28.
2. ISO group: Rats received two injections of ISO (85 mg/kg/day, s.c.) in a volume of 1.5 mL/kg, one injection per day at days 27 and 28 to induce acute MI [8,90,91,92]
3. FLP (15 mg/kg) + ISO group: Rats were treated with FLP at a dose of (15 mg/kg/day, p.o.) for 28 days, followed by two injections of ISO (85 mg/kg/day, s.c.), one injection per day at day 27 and 28.
4. FLP (30 mg/kg) + ISO group: Rats were treated with FLP at a dose of (30 mg/kg/day, p.o.) for 28 days, followed by two injections of ISO (85 mg/kg/day, s.c.), one injection per day at day 27 and 28.
5. FLP (45 mg/kg) + ISO group: Rats were treated with FLP at a dose of (45 mg/kg/day, p.o.) for 28 days, followed by two injections of ISO (85 mg/kg/day, s.c.), one injection per day on days 27 and 28.

### 4.4. Induction of MI

Isoproterenol (85 mg/kg) injected s.c. at 24 h intervals for two successive days was previously demonstrated to Induce biochemical, histopathological, and ECG changes characteristics for MI [8,90,91,92].

### 4.5. Electrocardiogram Monitoring

After 24 h of the second ISO dose, ECG for all groups was recorded. Rats were anesthetized by inhaling tetrahydrofuran until complete anesthesia was reached. ECG electrodes were then affixed subcutaneously to the paws of the rats while they were lying on their backs and then connected to the Kaden Yasen™ ECG-903 device (Zhuhai City, Guangdong, China). The following ECG parameters were analyzed: HR, R wave amplitude, ST segment amplitude, and Qt interval.

### 4.6. Collection of Blood and Tissue Samples

After ECG recordings, Under the light tetrahydrofuran anesthesia, blood samples were taken from the orbital sinus (retro-orbital plexus) using a clean, sterile capillary tube that was introduced into the inner canthus of the eye. Rats were then sacrificed by cervical dislocation, and the blood was left to be clotted for 20 min. Then, the samples were processed by centrifugation at 4000 rpm for 15 min. Before being used to measure heart damage indicators and 5-HT levels, serum samples were separated, collected in dry, clean tubes, and stored at −80. After sacrifice, hearts were cut free of connective tissue, cleaned in ice-cold phosphate-buffered saline, and finally weighed. The left ventricles were dissected and weighted to determine the ratios of heart weight to body weight and left ventricular weight to heart weight. These ratios were identified as heart hypertrophy indices.

Left ventricles were fixed in 10 % neutral buffered formalin for histopathological examination, while the remaining parts of the hearts were stored at −80 °C. Samples from the frozen hearts were homogenized in phosphate-buffered saline (10 % *w*/*v*) by means of Teflon homogenizer. Tissue homogenates were used to assay oxidative stress markers, 5-HT, and Ca^2+^. Other samples from the frozen hearts were used for Real-Time Quantitative Polymerase Chain Reaction (RT-qPCR) of 5-HT2A receptors.

### 4.7. Histopathological Examination of the Heart Tissue

The isolated left ventricles were first fixed in 10% neutral buffered formalin, then underwent conventional tissue processing, including dehydration in a succession of alcohol concentrations (70%, 95%, and 100%), clearing in xylene for 15 min, and embedding in paraffin for an hour. After being divided into 4 m slices and stained with hematoxylin and eosin (H&E), the paraffin-embedded sections were viewed under a light microscope. The changes in the ventricular histopathological parameters were scored as follows: (0) showing no changes, (1) showing mild changes, (2) showing moderate changes, and (3) showing severe changes [93,94].

### 4.8. Determination of Serum Cardiac Markers

Utilizing a UV-visible spectrophotometer (UV-1601PC, Schimadzu, Japan), the activities of CK and LDH, as well as CK-MB activities, were estimated using an Immunological ultraviolet assay. Moreover, cardiac TnI concentration was determined via an electrochemiluminescence sandwich immunoassay technique. All were performed according to the manufacturer’s instructions using commercial kits (Stanbio, Boerne, TX, USA).

### 4.9. Determination of the Cardiac Oxidative Stress Markers

Cardiac oxidative stress markers, including MDA and GSH, were determined using a spectrophotometric kit following the kit manufacturer protocol.

### 4.10. Determination of the Serum, Cardiac 5-HT, and the Cardiac Total Ca^2+^

Enzyme-linked immunosorbent assay (ELISA) was used for the determination of cardiac and serum levels of 5-HT. The assay was carried out according to the manufacturer’s instructions using an automated ELISA reader. Cardiac total Ca^2+^ concentration was determined using ultraviolet–visible spectrophotometer following the kit manufacturer protocol.

### 4.11. Determination of the Cardiac 5-HT2A Receptors Regulation

Polymerase chain reaction assay (PCR) was used to evaluate Cardiac 5-HT2A receptors gene expression (MGI:MGI:109521). Primer was designed, and the β-actin gene was used as an internal standard. Three biological replicates and for, every three technical replicates are being used. After the mRNA was extracted, it was tested for quality and quality by a nanodrop. A cDNA (5 μL) was used for qRT-PCR and was conducted using a Rotor-Gene thermocycler and SYBR Green I Master Mix, according to the manufacturer’s protocol, under the following PCR conditions: 95 °C for 1 min, followed by 40 cycles at 95 °C for 15 s, 60 °C for 15 s and 72 °C for 20 s. The relative expression marker gene was calculated using the 2^− ΔΔCT^ comparative CT method [95].

### 4.12. Statistical Analysis

The means and standard deviations were used to express all the data. One-way analysis of variance (ANOVA) was used to assess the statistical significance using SPSS (version 26, Chicago, IL, USA), and Duncan’s multiple range test (DMRT) was used to determine individual comparisons at *p* < 0.001. Non-parametric data were analyzed using Kruskal-Wallis, followed by Dunn’s test for multiple comparisons at *p* < 0.05. * *p* value < 0.05, ** *p* < 0.01, *** *p* < 0.001, *n* = 5.

## Figures and Tables

**Table 1:** Effect of pretreatment with FLP (15, 30, and 45 mg/kg) on the final body weight, heart weight, left ventricular weight, myocardial weight index, and left ventricle weight index in ISO myocardial infarct rats.

Group		Initial Body Weight (g)	Final Body Weight (g)	Heart Weight (g)	Left Ventricular Weight (g)	Myocardial Weight Index	Left Ventricle Weight Index
		Mean ± SD	Mean ± SD	Mean ± SD	Mean ± SD	Mean ± SD	Mean ± SD
Normal		123.13 ± 15.76 c	133.21 ± 17.55 b	0.51 ± 0.09 b	0.18 ± 0.03 c	0.38 ± 0.01 c	35.13 ± 0.62 b
ISO		137.12 ± 4.32 bc	149.78 ± 4.29 ab	0.70 ± 0.02 a	0.29 ± 0.02 a	0.47 ± 0.03 a	42.09 ± 0.94 a
FLP (15 mg/kg) + ISO		151.30 ± 5.14 ab	162.93 ± 5.55 a	0.71 ± 0.04 a	0.29 ± 0.03 a	0.44 ± 0.02 ab	40.58 ± 2.23 a
FLP (30 mg/kg) + ISO		162.41 ± 17.94 a	173.48 ± 19.04 a	0.71 ± 0.09 a	0.26 ± 0.04 ab	0.41 ± 0.01 bc	37.19 ± 0.98 b
FLP (45 mg/kg) + ISO		154.13 ± 4.26 ab	163.44 ± 4.76 a	0.64 ± 0.01 a	0.23 ± 0.01 b	0.39 ± 0.01 c	35.92 ± 1.00 b
ANOVA	F	5.708	4.877	6.491	9.965	11.89	16.715
	*p*	0.012 *	0.019 *	0.008 **	0.002 **	0.001 ***	<0.001 ***

Means followed by different letters are significantly different according to DMRT. *, **, *** significant at *p* < 0.05, 0.01, and 0.001. NS, non-significant at *p* > 0.05.

**Table 2:** Effect of pretreatment with FLP (15, 30, and 45 mg/kg) on serum and cardiac 5-HT level, in addition to cardiac GSH, MDA, and total Ca^2+^ concentrations in ISO myocardial infarct rats.

Group		5-HT (ng/mL-Serum)	5-HT (ng/g-Tissue)	Total Ca^2+^ (mg/dL-Tissue)
		Mean ± SD	Mean ± SD	Mean ± SD
Normal		87.90 ± 9.02 a	39.70 ± 1.55 a	1.7 ± 0.03 c
ISO		33.00 ± 2.26 e	15.40 ± 1.10 e	2.0 ± 0.05 a
FLP (15 mg/kg) + ISO		43.60 ± 6.70 d	23.80 ± 0.70 d	1.8 ± 0.04 b
FLP (30 mg/kg) + ISO		66.90 ± 4.02 c	28.70 ± 0.80 c	1.8 ± 0.03 b
FLP (45 mg/kg) + ISO		77.50 ± 2.25 b	33.40 ± 0.04 b	1.8 ± 0.06 c
ANOVA	F-ratio	52.024	269.747	18.74
	*p*-value	<0.001 ***	<0.001 ***	<0.001 ***

Means followed by different letters are significantly different according to DMRT. *** significant at 0.001. NS, non-significant at *p* > 0.05.

**Table 3:** Effect of pretreatment with FLP (15, 30, and 45 mg/kg) on cardiac 5-HT2A receptors CT, ΔCt, ΔΔCT, and normalized Fold change in ISO myocardial infarct rats.

Group		5-HT2A Receptors CT	5-HT2A Receptors ΔCt	5-HT2A Receptors ΔΔCT	5-HT2A Receptors Normalized Fold Change
		Mean ± SD	Mean ± SD	Mean ± SD	Mean ± SD
Normal		28.53 ± 0.55 a	4.62 ± 0.05 a	0.00 ± 0.00 a	0.80 ± 0.40 d
ISO		23.23 ± 0.55 d	2.98 ± 0.06 e	−1.64 ± 0.04 e	2.92 ± 0.40 a
FLP (15 mg/kg) + ISO		24.70 ± 0.55 c	3.38 ± 0.06 d	−1.24 ± 0.04 d	2.16 ± 0.40 b
FLP (30 mg/kg) + ISO		25.46 ± 0.55 bc	3.68 ± 0.06 c	−0.94 ± 0.04 c	1.72 ± 0.40 bc
FLP (45 mg/kg) + ISO		26.07 ± 0.55 b	4.00 ± 0.05 b	−0.62 ± 0.02 b	1.34 ± 0.40 cd
ANOVA	F-ratio	37.816	383.855	1119.577	12.026
	*p*-value	<0.001 ***	<0.001 ***	<0.001 ***	<0.001 ***

Means followed by different letters are significantly different according to DMRT. *** significant at 0.001. NS, non-significant at *p* > 0.05.

## Data Availability

Data is contained within the article.
